# Supplementary material for: Surface Microbiome Profiling of Dental Elevators Using Third‐Generation Sequencing: Implications for Infection Control in Dental Practice
Source: Microbiologyopen. 2025 Dec 8;14(6):e70178. doi: 10.1002/mbo3.70178 (PMC12685768; doi:10.1002/mbo3.70178)
Supplement: Supplementary file 1 — customer_backup. [file MBO3-14-e70178-s001.zip › customer_backup/customer_backup/taxa_summary/krona/groups/treat/M09.Krona.html]

Javascript must be enabled to view this page.

members
magnitude
magnitudeUnassigned

M09.krona

60723

60421

3

3

3

2

2

2

0

0

0

1

1

1

0

0

0

0

0

0

0

0

0

0

0

0

0

0

0

0

0

1

1

1

1

0

0

0

0

0

0

0

0

1

1

25

21

0

0

0

0

1

1

1

1

0

0

0

0

0

0

0

0

0

20

1

1

1

1

0

0

1

1

2

2

2

0

0

14

14

14

2

1

1

0

0

0

0

1

1

0

0

0

0

0

0

4

0

0

0

0

0

0

0

4

0

0

0

1

1

1

0

0

0

0

0

0

3

3

3

132

35

35

0

0

0

35

9

9

0

26

26

0

0

0

0

0

30

1

1

1

1

29

22

22

22

0

0

0

0

0

0

0

0

0

0

7

1

0

1

0

0

0

0

0

5

1

2

2

0

1

1

0

0

0

0

0

0

0

0

5

5

5

5

5

0

0

53

53

22

0

0

3

3

1

1

0

0

6

6

0

0

12

12

0

2

2

2

0

0

2

2

2

27

15

1

13

0

1

12

12

9

9

8

2

2

6

6

1

1

1

0

0

0

0

0

0

0

0

0

0

0

0

0

0

0

0

0

0

0

0

0

0

0

1

1

1

1

1

1

0

0

0

0

0

0

0

0

0

0

0

0

0

0

0

0

0

0

0

0

0

0

0

0

0

0

0

0

0

0

0

0

25

0

0

0

0

0

0

0

0

0

0

0

0

0

2

2

2

2

2

23

23

23

0

0

0

23

13

0

7

0

3

0

0

0

0

0

79

17

17

0

0

0

17

17

17

52

52

0

0

0

0

0

0

52

52

52

0

0

0

0

0

10

0

0

0

0

0

0

0

0

0

0

0

0

0

0

0

0

0

0

0

0

0

0

0

0

10

0

0

0

0

0

0

0

0

3

0

0

2

2

1

1

1

1

0

1

0

0

0

0

0

6

6

1

5

0

0

0

0

0

0

0

0

0

0

0

0

0

0

0

0

0

0

27609

5286

4

4

4

4

2

0

0

0

2

1

1

0

0

0

0

0

0

1

0

1

0

0

0

0

7

7

7

1

6

0

0

0

0

0

0

0

0

0

0

0

0

0

0

0

0

0

0

0

0

0

0

0

0

0

0

0

0

0

0

0

1

1

1

1

0

0

0

83

83

0

0

2

1

0

0

0

1

0

0

0

0

0

0

0

0

0

0

0

0

0

0

0

0

0

0

0

0

0

0

0

0

3

0

3

0

0

0

0

0

78

0

0

12

0

0

0

3

0

0

0

0

0

1

0

0

0

22

2

14

0

3

0

16

1

0

2

0

0

2

0

0

0

0

0

0

1

0

0

0

1

0

0

1

1

5128

3

0

0

0

0

0

0

0

2

0

2

1

1

0

0

0

0

0

0

0

0

44

0

0

0

44

0

36

0

0

8

0

0

0

0

0

0

0

0

0

0

0

0

0

0

0

0

0

0

0

0

4399

1

1

0

0

3

3

0

319

319

0

11

11

0

3052

3052

2

1

1

0

0

0

0

769

0

12

757

0

0

0

0

0

0

242

0

0

0

0

0

1

0

0

0

1

1

239

0

0

0

0

0

0

0

0

0

0

0

0

0

0

0

0

0

0

0

0

0

0

0

0

0

7

7

7

0

0

0

0

0

0

0

0

0

0

0

0

14

13

3

10

1

1

0

106

0

0

0

0

0

3

3

0

0

0

0

0

103

103

0

0

0

0

0

0

0

0

0

0

0

555

0

0

0

0

0

0

0

0

0

0

0

0

3

0

0

0

0

2

0

0

0

1

0

0

0

0

0

0

0

8

8

0

0

0

0

0

0

0

0

0

544

544

0

0

0

0

0

0

11

0

0

0

0

0

11

0

0

0

0

0

0

0

0

0

0

0

0

0

0

0

11

11

0

0

0

0

0

0

1

1

0

0

0

0

1

1

0

0

0

0

0

0

0

0

0

0

0

0

0

0

0

0

0

0

0

0

0

0

0

0

0

0

0

0

0

0

0

0

0

0

0

0

0

0

0

13

13

0

0

0

0

0

11

11

1

0

0

0

0

0

1

0

0

0

0

0

0

0

0

0

0

1

0

0

1

0

0

0

0

0

0

0

0

4

0

0

0

1

1

1

0

0

0

0

0

0

3

3

3

0

0

0

0

0

0

0

0

0

0

0

0

31

31

0

0

0

0

0

0

0

0

2

2

0

0

0

1

0

0

1

0

0

0

0

1

1

0

0

0

0

0

0

0

0

0

15

0

0

0

0

15

1

0

0

1

0

0

0

0

11

0

11

0

22323

0

0

0

0

0

0

0

0

0

0

0

0

1

1

1

1

0

0

0

14071

123

0

0

123

123

29

29

29

0

0

0

0

0

0

0

0

0

0

0

0

0

0

0

0

0

0

0

0

0

0

1

1

1

0

0

7848

0

0

0

0

7833

7833

11

2

2

5

2

4

0

0

0

0

0

2

0

0

0

1

0

0

1

0

0

0

0

0

0

0

0

0

0

0

0

0

0

0

0

0

0

0

0

0

0

2

0

0

0

0

1

1

0

0

0

0

0

0

0

0

1

1

51

11

11

0

0

0

0

0

40

0

40

0

1

1

1

5922

0

0

0

0

0

0

0

0

666

666

0

0

2

0

0

2

5

0

5

0

0

142

0

142

0

0

1

0

1

262

0

262

0

0

0

3989

0

3989

854

854

1

1

94

0

0

94

0

94

40

0

0

0

0

0

0

0

0

40

40

9

31

0

0

0

0

0

0

0

0

12

6

6

6

6

6

6

8

8

8

8

0

0

0

0

0

3

2

2

1

1

0

1

1

1

0

0

0

0

0

0

4380

0

0

0

2

0

0

0

0

0

0

0

0

0

2

0

0

0

2

1

1

1

0

0

0

0

0

0

0

0

0

0

0

0

0

0

0

0

0

0

4344

4344

65

0

3779

500

33

20

1

19

0

0

0

0

9

4

2

3

3

3

1

1

0

0

0

0

0

0

0

0

0

0

0

0

0

0

0

0

0

0

0

0

0

0

0

0

0

0

6

4

4

4

2

0

0

0

0

2

1

1

6

6

6

6

1881

9

4

4

5

5

2

2

2

12

0

0

0

12

12

0

0

0

1796

80

80

7

7

1709

1709

0

0

2

2

2

0

11

11

11

0

0

0

36

36

36

13

4

4

0

0

3

3

0

0

0

0

0

0

0

6

6

0

1

1

1

1

7

0

0

0

7

7

7

0

0

0

0

0

0

0

0

0

0

0

0

0

0

0

0

0

0

0

0

0

0

1831

2

0

0

0

0

0

1

1

0

0

1

0

1

1829

1824

1824

1

0

1

0

0

0

0

0

0

0

0

0

4

0

1

0

0

0

0

0

0

0

0

1

0

2

0

0

0

0

0

0

0

2

2

2

2

0

0

0

0

0

0

0

0

0

0

0

19

19

0

0

0

0

1

1

0

0

5

5

0

0

13

3

1

9

0

0

0

0

0

0

0

0

0

0

35

0

0

0

1

1

1

34

34

34

15

15

4

1

3

11

11

5

5

5

5

0

33

10

10

0

0

0

10

0

0

0

0

0

0

0

0

9

0

9

0

0

0

0

1

0

1

0

23

0

0

0

0

0

0

0

0

0

0

0

0

0

0

0

0

0

23

0

0

0

0

0

0

0

0

0

0

0

0

0

0

0

0

0

0

0

0

0

23

0

0

0

23

13

0

10

0

0

0

0

0

0

0

0

0

0

0

0

0

0

0

0

0

0

0

0

0

0

0

0

0

0

0

0

0

0

0

0

0

0

0

0

0

0

0

0

0

0

0

0

0

0

0

0

0

0

0

0

0

9950

0

0

0

0

0

9948

19

0

0

0

1

0

0

0

0

0

0

0

0

0

0

0

0

0

0

0

0

1

1

0

0

0

0

0

0

0

0

0

0

0

0

0

0

0

0

0

0

0

0

0

0

0

0

0

0

0

0

0

0

0

0

0

0

0

0

18

18

18

0

0

0

0

0

1726

0

0

0

59

1

1

0

0

4

4

0

0

36

1

21

14

6

6

1

1

1

1

0

0

0

2

0

0

2

0

0

0

0

1

1

0

0

2

2

0

0

0

0

4

4

1

1

0

0

5

5

5

0

7

0

0

7

7

1655

0

0

0

0

1530

1530

125

125

8192

1

0

0

1

1

60

60

60

0

0

0

6225

0

0

27

27

787

787

5411

5411

400

0

0

0

0

0

0

0

0

0

0

0

0

0

400

7

0

5

0

0

0

0

317

49

22

31

31

31

4

0

0

0

0

4

0

0

4

4

1

1

3

3

21

21

21

945

945

0

1

6

2

8

135

1

0

1

0

679

0

1

107

0

1

3

96

0

0

58

9

49

38

13

0

1

21

0

3

405

8

2

6

189

0

189

50

0

37

13

0

0

107

107

20

20

0

0

16

0

0

0

0

1

0

0

0

0

0

0

0

0

0

0

0

15

15

0

0

0

0

10

0

0

0

0

0

4

0

0

1

0

0

0

0

0

0

0

0

0

0

0

0

0

0

0

0

0

0

0

0

0

0

1

0

0

0

0

0

0

0

0

0

0

0

0

0

0

0

0

0

0

0

0

0

0

1

1

1

10

0

0

0

0

0

0

0

0

0

0

0

2

0

0

2

0

2

0

0

0

0

0

0

0

0

0

0

0

0

0

0

0

0

0

0

0

0

0

0

0

0

0

0

0

0

0

0

8

0

0

0

0

0

0

0

0

0

1

1

0

0

0

0

0

0

7

0

0

0

0

0

0

2

0

0

3

1

0

1

0

0

0

0

0

1

1

1

0

0

1

1

0

0

0

0

0

0

0

0

1

1

0

0

0

0

1

1

1

0

0

0

0

0

0

2

2

1

1

0

0

1

0

1

0

0

0

0

0

0

0

0

0

0

0

0

0

0

0

0

0

0

0

0

1

1

1

1

0

0

0

0

0

0

0

0

0

0

0

0

0

0

0

0

0

0

0

0

0

0

4

4

4

4

4

4

0

0

1164

223

223

2

2

2

58

19

19

0

0

0

39

0

18

21

0

163

0

0

151

18

25

12

96

0

0

0

0

0

0

0

0

0

6

1

5

1

0

0

1

5

0

0

5

0

0

2

2

2

2

2

87

35

17

0

0

2

2

3

3

0

0

2

2

8

8

2

2

18

18

0

18

6

0

0

0

5

5

5

0

0

0

0

0

0

0

0

0

1

1

1

2

0

0

0

0

0

0

0

0

0

0

0

0

2

2

2

0

0

0

0

0

0

0

44

10

0

0

0

0

0

0

10

10

0

0

0

0

0

0

0

31

0

0

0

28

0

28

3

2

1

3

0

0

0

0

3

3

50

48

0

0

0

24

22

10

5

0

1

1

5

0

0

0

0

0

0

2

0

1

1

0

0

0

0

0

0

24

1

1

0

0

23

23

0

0

0

0

2

2

2

1

1

0

0

0

0

0

0

0

0

801

1

1

1

1

0

0

0

0

0

0

0

0

0

0

0

0

0

0

0

0

2

2

0

0

0

0

0

0

0

1

1

0

0

0

0

0

0

0

0

0

1

0

1

0

0

0

0

0

0

0

0

0

1

1

1

0

0

1

0

0

0

0

258

37

27

0

17

7

3

10

10

0

0

0

0

0

0

221

204

19

185

0

0

17

17

0

0

174

174

17

17

0

157

33

71

31

0

0

13

0

9

0

0

16

0

0

0

0

0

0

0

1

0

0

1

1

0

0

0

0

0

0

0

0

0

0

0

15

0

0

1

1

14

14

0

0

0

0

0

0

0

0

0

0

0

0

0

27

27

9

9

0

0

18

1

0

0

0

0

3

8

0

0

0

3

2

1

3

3

3

3

0

0

0

0

0

0

0

0

0

0

0

0

0

0

0

98

97

1

1

0

0

0

0

0

0

4

4

0

0

0

1

0

1

91

0

91

0

0

0

1

0

0

0

0

0

0

1

0

0

0

0

0

0

0

1

0

0

0

0

0

0

0

0

0

0

0

0

0

0

0

0

0

0

0

0

0

0

3

3

0

0

0

0

3

3

0

0

0

0

0

0

0

0

0

0

0

0

0

0

0

0

0

0

0

0

0

0

0

218

162

14

14

1

1

1

0

1

0

0

141

141

0

4

0

2

0

0

2

0

0

0

0

1

1

1

0

0

0

0

0

0

0

0

0

0

0

0

1

1

0

0

0

0

0

2

2

2

0

0

0

0

52

1

1

0

0

0

0

0

0

0

0

0

0

0

0

0

0

0

0

0

0

0

0

0

0

0

51

0

43

8

0

0

0

0

0

0

0

0

0

0

0

1

1

1

0

0

1

1

1

1

0

1

0

0

19

19

19

19

0

0

19

5

0

0

0

0

12

0

0

0

0

0

0

0

0

0

0

0

0

0

2

0

0

0

0

0

0

0

0

0

0

0

0

0

0

0

0

0

7

7

7

7

7

7

144

144

144

64

0

0

11

11

1

1

52

38

14

80

80

80

0

0

0

0

0

0

0

0

139

139

139

39

39

0

0

39

0

0

82

82

0

82

0

1

1

1

0

0

0

17

17

17

0

0

0

0

0

0

0

0

0

0

0

0

0

0

0

0

0

0

0

0

0

0

0

0

0

0

0

1

1

1

1

1

1

0

0

0

29

5

0

0

0

0

5

1

1

1

0

0

0

0

0

0

0

4

0

0

0

0

0

0

4

4

2

2

2

2

0

1

1

0

0

0

0

0

0

0

0

0

0

0

0

0

0

0

0

0

0

0

0

0

0

0

0

0

0

0

0

0

0

0

0

0

0

0

0

0

0

0

0

11

11

11

3

3

8

0

2

0

0

0

0

0

6

1

1

1

1

1

10

10

9

9

9

0

0

0

1

1

1

0

0

0

0

229

0

0

0

0

0

0

0

0

0

0

0

0

0

1

0

0

0

0

0

0

0

0

0

0

0

0

0

0

0

1

1

1

1

0

0

0

0

0

0

0

0

0

0

0

0

0

0

0

0

0

0

0

0

0

0

0

0

0

0

0

0

0

0

0

0

0

0

0

228

228

0

0

0

0

0

0

42

42

42

0

0

0

0

0

0

0

0

186

101

101

2

0

2

24

24

0

0

47

47

12

12

0

0

0

0

0

0

0

0

0

0

0

0

0

0

0

0

0

0

0

0

0

87

87

87

87

0

0

87

13

74

0

0

0

0

0

0

0

0

0

0

0

0

0

0

0

0

0

0

0

0

0

0

0

0

0

0

0

0

0

0

0

0

0

0

0

0

0

0

0

0

0

0

0

0

0

0

0

0

0

0

0

0

0

0

0

9

9

9

9

9

0

8

1

0

0

0

0

0

0

0

0

0

0

0

0

0

0

0

0

0

0

0

0

20437

13526

8

8

0

0

0

8

8

0

0

0

0

0

0

0

104

104

0

0

104

104

2

1

1

1

0

0

0

1

1

1

0

0

79

79

79

12

13

54

11163

11163

5

5

0

0

0

0

0

0

0

80

21

59

0

0

0

164

103

61

0

0

0

1

1

0

0

0

31

31

20

20

0

0

0

0

6782

6696

77

9

13

1

0

12

0

0

88

5

1

4

0

3

0

0

6

0

44

1

0

0

23

0

1

0

0

0

0

0

0

0

0

36

18

18

0

0

66

1

55

10

22

3

3

0

9

0

7

1408

13

11

0

37

958

53

333

3

0

10

10

947

0

9

51

848

39

246

246

0

0

58

58

0

0

0

0

0

21

0

21

0

0

42

42

0

0

108

0

17

90

1

0

0

0

0

89

89

2

2

0

0

0

0

0

0

25

25

105

5

100

0

0

0

794

0

511

3

26

2

220

9

8

0

0

2

13

0

0

0

0

0

0

0

0

0

0

315

95

13

13

0

0

0

0

0

0

0

0

0

40

40

0

0

0

0

0

0

0

0

0

42

42

0

0

0

204

3

3

0

0

39

39

0

0

0

0

0

0

162

162

0

16

0

0

0

0

0

0

0

0

0

16

16

0

0

45

45

0

0

0

0

20

18

0

1

0

0

0

1

25

25

74

6

6

6

0

0

0

0

0

0

12

12

12

56

56

56

0

0

0

0

0

0

0

0

0

1572

1015

95

0

95

0

0

3

3

15

0

15

138

138

0

0

20

1

0

19

0

0

0

0

0

1

0

0

1

0

81

1

20

0

60

0

342

156

0

1

0

114

33

0

0

38

0

0

0

14

14

23

3

20

283

67

1

0

215

39

39

39

0

0

0

25

9

9

0

16

16

0

0

0

0

0

0

0

0

23

23

23

0

0

0

0

0

470

14

0

0

0

0

0

14

0

0

1

1

139

117

22

0

47

0

47

0

0

0

0

0

213

212

1

41

41

15

0

15

0

0

0

164

57

57

57

27

27

27

71

71

71

0

0

0

9

9

9

5634

0

0

0

0

214

18

0

0

0

0

0

0

4

0

3

0

1

0

1

0

1

12

12

1

1

0

0

0

0

196

0

0

0

0

0

0

0

196

0

0

1

0

2

4

1

0

0

1

21

60

2

0

1

73

30

0

0

0

0

20

20

19

0

19

0

0

1

1

2662

208

0

0

0

0

35

35

164

5

1

0

157

1

0

0

0

1

1

1

0

1

7

7

2454

52

52

629

629

2

2

0

0

0

0

1409

1406

3

1

1

0

0

13

13

59

0

59

29

29

259

259

1

1

0

0

0

0

0

0

0

0

0

0

0

0

108

81

0

0

0

0

81

63

18

27

27

26

0

1

1

1

1

0

1

22

22

0

0

0

0

22

0

0

0

1

1

0

9

0

0

0

0

0

0

0

1

0

0

0

0

4

0

0

0

0

0

6

0

0

0

0

0

0

0

0

222

19

19

19

0

0

0

85

85

85

118

118

118

1

1

1

1

0

2361

8

8

7

1

10

0

0

0

0

10

10

0

0

0

0

340

334

1

39

13

0

148

22

29

36

32

2

12

0

0

6

6

0

5

5

1

4

1982

0

0

4

4

30

11

8

11

11

0

3

8

718

4

270

9

0

0

435

8

8

805

648

0

0

1

0

156

21

21

372

1

1

370

8

2

6

2

2

3

3

0

1

1

1

15

14

14

0

0

1

1

2

2

2

0

0

0

0

2

0

21

21

0

0

21

21

0

0

0

0

0

0

0

0

0

0

0

0

0

0

0

0

0

1277

0

0

0

0

0

0

1277

1

1

1

289

0

0

24

24

3

3

0

0

0

262

62

118

0

1

0

70

0

11

0

986

922

922

0

22

22

8

6

0

0

2

34

33

1

1

1

1

0

0

0

0

0

7

0

0

0

0

0

0

0

0

0

0

0

0

0

0

0

0

0

0

0

0

0

0

0

0

4

0

0

0

0

0

0

0

0

0

0

0

4

4

4

4

2

0

0

0

0

2

2

1

0

1

0

0

0

1

1

0

0

0

0

0

0

0

0

0

0

0

0

0

0

0

0

0

0

0

0

0

0

0

0

0

1

1

1

1

1

285

285

285

285

285

0

285

0

0

0

0

0

0

0

0

0

0

0

0

0

0

0

0

0

0

0

0

0

0

0

0

0

0

0

0

0

0

0

0

0

0

24

24

24

24

24

24

24

278

79

79

79

10

10

10

69

69

69

0

0

0

0

0

0

0

0

195

195

195

195

195

195

4

4

4

4

4

4
